# Propane-1,3-diammonium dichromate(VI)

**DOI:** 10.1107/S1600536812031042

**Published:** 2012-07-14

**Authors:** Sonia Trabelsi, Houda Marouani, Salem S. Al-Deyab, Mohamed Rzaigui

**Affiliations:** a Laboratoire de Chimie des Matériaux, Faculté des Sciences de Bizerte, 7021 Zarzouna Bizerte, Tunisia; b Petrochemical Research Chair, College of Science, King Saud University, Riyadh, Saudi Arabia

## Abstract

The title compound, (C_3_H_12_N_2_)[Cr_2_O_7_], consists of a discrete dichromate anion with an eclipsed conformation and a propane-1,3-diammonium cation. Both kinds of ions have a mirror plane passing through the bridging O atom and the central methyl­ene C atom of the Cr_2_O_7_^2−^ and C_3_H_12_N_2_^2+^ moieties, respectively. Anions and cations are alternately stacked to form columns parallel to the *b* axis. Ions are linked by intra- and inter-column hydrogen bonds of types N—H⋯O and C—H⋯O, involving O atoms of the dichromate anions as acceptors, and ammonium or methyl­ene groups as donors.

## Related literature

For related structures, see: Akriche & Rzaigui (2009 ▶); Sieroń (2007 ▶); Khadhrani *et al.* (2006 ▶); Kallel *et al.* (1980 ▶); Pritchard *et al.* (1992 ▶). For a discussion on hydrogen bonding, see: Brown (1976 ▶); Blessing (1986 ▶). For background on Cr^VI^ species as industrial waste, see: Wani *et al.* (2007 ▶).
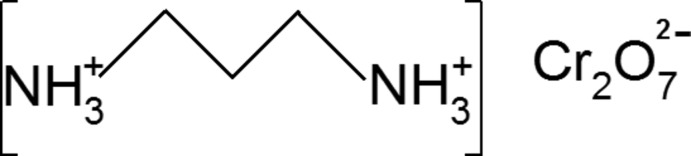


## Experimental

### 

#### Crystal data

(C_3_H_12_N_2_)[Cr_2_O_7_]
*M* *_r_* = 292.15
Orthorhombic, 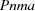

*a* = 8.818 (2) Å
*b* = 13.764 (2) Å
*c* = 7.918 (2) Å
*V* = 961.1 (4) Å^3^
*Z* = 4
Ag *K*α radiation
λ = 0.56083 Å
μ = 1.18 mm^−1^
*T* = 293 K
0.30 × 0.15 × 0.10 mm

#### Data collection

Enraf–Nonius CAD4 diffractometer
4877 measured reflections
2430 independent reflections
1811 reflections with *I* > 2σ(*I*)
*R*_int_ = 0.020
2 standard reflections every 120 min intensity decay: 3%

#### Refinement

*R*[*F* ^2^ > 2σ(*F* ^2^)] = 0.032
*wR*(*F* ^2^) = 0.096
*S* = 1.10
2430 reflections
69 parameters
H-atom parameters constrained
Δρ_max_ = 0.79 e Å^−3^
Δρ_min_ = −0.61 e Å^−3^

### 

Data collection: *CAD-4 EXPRESS* (Enraf–Nonius, 1994 ▶); cell refinement: *CAD-4 EXPRESS*; data reduction: *XCAD4* (Harms & Wocadlo, 1995 ▶); program(s) used to solve structure: *SIR92* (Altomare *et al.*, 1994 ▶); program(s) used to refine structure: *SHELXL97* (Sheldrick, 2008 ▶); molecular graphics: *ORTEP-3* (Farrugia, 1997 ▶) and *DIAMOND* (Brandenburg & Putz, 2005 ▶); software used to prepare material for publication: *WinGX* (Farrugia, 1999 ▶).

## Supplementary Material

Crystal structure: contains datablock(s) I, global. DOI: 10.1107/S1600536812031042/bh2445sup1.cif

Structure factors: contains datablock(s) I. DOI: 10.1107/S1600536812031042/bh2445Isup2.hkl

Additional supplementary materials:  crystallographic information; 3D view; checkCIF report

## Figures and Tables

**Table 1:** Hydrogen-bond geometry (Å, °)

*D*—H⋯*A*	*D*—H	H⋯*A*	*D*⋯*A*	*D*—H⋯*A*
N1—H1*A*⋯O3^i^	0.89	2.12	2.9609 (19)	156
N1—H1*B*⋯O2^ii^	0.89	1.99	2.8168 (19)	154
N1—H1*C*⋯O4	0.89	2.17	2.955 (2)	147
N1—H1*C*⋯O2^iii^	0.89	2.44	2.9844 (19)	120
C1—H1*D*⋯O3	0.97	2.51	3.405 (2)	153
C1—H1*E*⋯O2^iv^	0.97	2.59	3.176 (2)	119

Symmetry codes: (i) 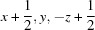; (ii) 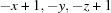; (iii) 
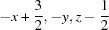
; (iv) 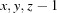.

**Fig. 1.**
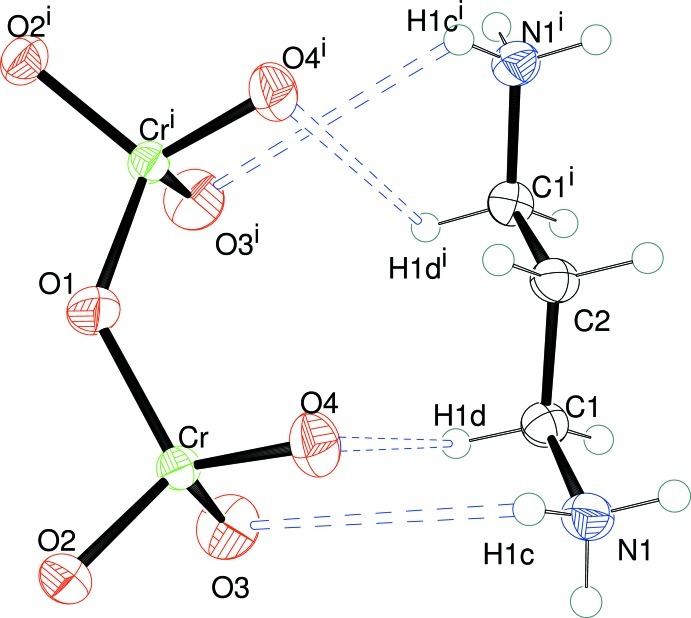
An ORTEP view of the title compound with displacement ellipsoids at the 30% probability level. H atoms are represented as small spheres of arbitrary radii. Symmetry code: (i) x, y-1, z

**Fig. 2.**
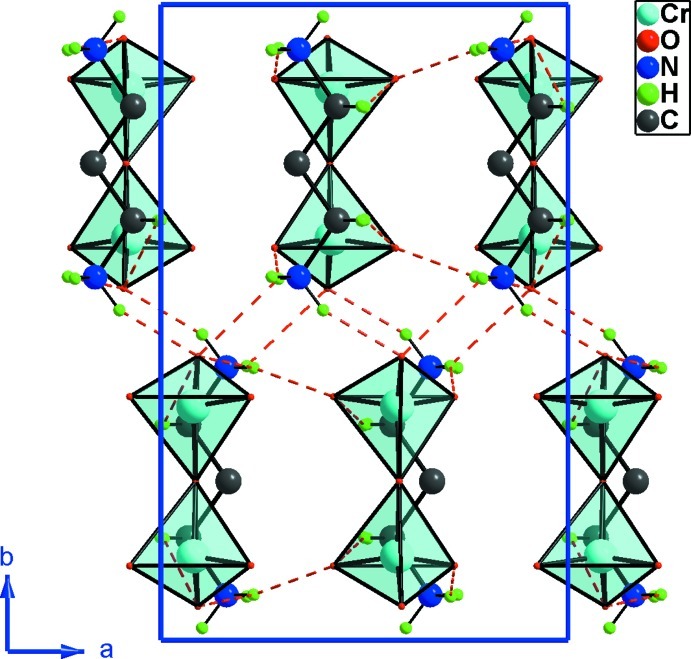
Projection of the crystal structure along the c axis.

## supplementary crystallographic information

### Comment

Hexavalent chromium is a predominant waste product of several metal finishing, petroleum refining and steel industries (Wani *et al.*, 2007). It exists as chromate in basic and neutral medium and as dichromate in acidic environment.

In presence of 1,3-diaminopropane in water, the chromic acid is condensed into dichromate to form the hybrid title compound, (C_3_H_12_N_2_)Cr_2_O_7_. The observed molecular structure is depicted in Fig. 1. To counter-balance the electric charge of Cr_2_O_7_^2-^, the used 1,3-diaminopropane has been doubly protonated. The title compound crystallizes in the orthorhombic *Pnma* space group, so that the dichromate anion and 1,3-diammoniumpropane should be symmetrical with respect to the symmetry plane (*m*). Owing of the passage of the latter through the bridging atoms O1 and C2 of Cr_2_O_7_ and C_3_H_12_N_2_ respectively, the asymmetric unit is built by one independent CrO_4_ group and the half of a 1,3-diammoniumpropane cation. The main geometrical features of Cr_2_O_7_^2-^ agree with those previously observed for this group in other compounds (Akriche & Rzaigui, 2009; Sieroń, 2007; Khadhrani *et al.*, 2006).

The bond lengths and the angles within the cation are comparable with those observed in other 1,3-diammoniumpropane salts such as [C_3_H_12_N_2_]ZnCl_4_ (Kallel *et al.*, 1980) and [C_3_H_12_N_2_](ClO_4_)_2_ (Pritchard *et al.*, 1992). In this structure, the cations and anions are alternately stacked to form columns parallel to the axis *b* (Fig. 2). The electrostatic interactions and H-bonds intra and inter columns keep up the three-dimensional network cohesion. The established weak H-bonds (Brown, 1976; Blessing, 1986) of types N—H···O and C—H···O involve oxygen atoms of the dichromate anions as acceptors, and the protonated nitrogen atoms and carbon atoms of 1,3-diammoniumpropane as donors.

### Experimental

Single crystals of the title compound were prepared at room temperature by dissolving CrO_3_ (0.10 g, 1 mmol) and 1,3-diaminopropane (0.07 g, 1 mmol) in distilled water (20 ml). The resulting solution was stirred during 30 min. and then evaporated slowly at room temperature until the formation of orange prismatic single crystals.

### Refinement

All H atoms attached to C and N atoms were fixed geometrically and treated as riding with C—H = 0.97 Å (methylene) and N—H = 0.89 Å. Isotropic displacement parameters for H atoms were calculated as *U*_iso_(H) = 1.2*U*_eq_(C) for CH_2_ groups and *U*_iso_(H) = 1.5*U*_eq_(N1) for the ammonium group.

### Crystal data

**Table:**

(C_3_H_12_N_2_)[Cr_2_O_7_]	*F*(000) = 592
*M**_r_* = 292.15	*D*_x_ = 2.019 Mg m^−^^3^
Orthorhombic, *P**n**m**a*	Ag *K*α radiation, λ = 0.56083 Å
Hall symbol: -P 2ac 2n	Cell parameters from 25 reflections
*a* = 8.818 (2) Å	θ = 9–11°
*b* = 13.764 (2) Å	µ = 1.18 mm^−^^1^
*c* = 7.918 (2) Å	*T* = 293 K
*V* = 961.1 (4) Å^3^	Prism, orange
*Z* = 4	0.30 × 0.15 × 0.10 mm

### Data collection

**Table:**

Enraf–Nonius CAD4 diffractometer	*R*_int_ = 0.020
Radiation source: fine-focus sealed tube	θ_max_ = 28.0°, θ_min_ = 2.3°
Graphite monochromator	*h* = −14→3
non–profiled ω scans	*k* = −23→3
4877 measured reflections	*l* = −3→13
2430 independent reflections	2 standard reflections every 120 min
1811 reflections with *I* > 2σ(*I*)	intensity decay: 3%

### Refinement

**Table:**

Refinement on *F*^2^	Secondary atom site location: difference Fourier map
Least-squares matrix: full	Hydrogen site location: inferred from neighbouring sites
*R*[*F*^2^ > 2σ(*F*^2^)] = 0.032	H-atom parameters constrained
*wR*(*F*^2^) = 0.096	*w* = 1/[σ^2^(*F*_o_^2^) + (0.0502*P*)^2^ + 0.243*P*] where *P* = (*F*_o_^2^ + 2*F*_c_^2^)/3
*S* = 1.10	(Δ/σ)_max_ = 0.001
2430 reflections	Δρ_max_ = 0.79 e Å^−^^3^
69 parameters	Δρ_min_ = −0.61 e Å^−^^3^
0 restraints	Extinction correction: *SHELXL97*, Fc^*^=kFc[1+0.001xFc^2^λ^3^/sin(2θ)]^-1/4^
0 constraints	Extinction coefficient: 0.024 (2)
Primary atom site location: structure-invariant direct methods	

### Fractional atomic coordinates and isotropic or equivalent isotropic displacement parameters (Å^2^)

**Table:**

	*x*	*y*	*z*	*U*_iso_*/*U*_eq_	
Cr	0.58026 (3)	0.131997 (16)	0.65346 (3)	0.01952 (8)	
O2	0.59121 (13)	0.05330 (8)	0.80635 (15)	0.0271 (2)	
O4	0.72115 (14)	0.11753 (9)	0.52532 (16)	0.0324 (3)	
O3	0.42366 (14)	0.11686 (10)	0.55239 (19)	0.0388 (3)	
O1	0.5853 (2)	0.2500	0.7434 (2)	0.0322 (4)	
N1	0.66136 (15)	0.07105 (9)	0.16706 (17)	0.0263 (2)	
H1A	0.7277	0.0716	0.0824	0.039*	
H1B	0.6016	0.0192	0.1578	0.039*	
H1C	0.7109	0.0687	0.2649	0.039*	
C2	0.6661 (2)	0.2500	0.1633 (3)	0.0237 (3)	
H2A	0.7327	0.2500	0.0658	0.028*	
H2B	0.7285	0.2500	0.2642	0.028*	
C1	0.56785 (16)	0.16037 (11)	0.16067 (19)	0.0231 (2)	
H1D	0.4995	0.1615	0.2567	0.028*	
H1E	0.5070	0.1601	0.0586	0.028*	

### Atomic displacement parameters (Å^2^)

**Table:**

	*U*^11^	*U*^22^	*U*^33^	*U*^12^	*U*^13^	*U*^23^
Cr	0.02104 (11)	0.01893 (11)	0.01860 (11)	−0.00012 (7)	−0.00096 (8)	0.00048 (7)
O2	0.0335 (5)	0.0232 (4)	0.0245 (4)	−0.0013 (4)	−0.0003 (4)	0.0043 (4)
O4	0.0308 (6)	0.0386 (6)	0.0277 (5)	0.0028 (4)	0.0079 (5)	0.0010 (5)
O3	0.0285 (6)	0.0481 (7)	0.0398 (7)	−0.0008 (5)	−0.0122 (5)	−0.0022 (6)
O1	0.0481 (10)	0.0202 (6)	0.0282 (7)	0.000	0.0002 (7)	0.000
N1	0.0279 (6)	0.0220 (5)	0.0289 (6)	0.0000 (5)	0.0022 (5)	0.0018 (5)
C2	0.0207 (8)	0.0216 (7)	0.0287 (9)	0.000	0.0028 (7)	0.000
C1	0.0206 (6)	0.0235 (6)	0.0252 (6)	−0.0010 (4)	−0.0002 (5)	−0.0005 (5)

### Geometric parameters (Å, º)

**Table:**

Cr—O3	1.6096 (13)	N1—H1C	0.8900
Cr—O4	1.6165 (13)	C2—C1	1.5077 (19)
Cr—O2	1.6274 (12)	C2—C1^i^	1.5077 (19)
Cr—O1	1.7740 (8)	C2—H2A	0.9700
O1—Cr^i^	1.7740 (8)	C2—H2B	0.9700
N1—C1	1.481 (2)	C1—H1D	0.9700
N1—H1A	0.8900	C1—H1E	0.9700
N1—H1B	0.8900		
			
O3—Cr—O4	109.35 (8)	C1—C2—C1^i^	109.83 (17)
O3—Cr—O2	109.55 (7)	C1—C2—H2A	109.7
O4—Cr—O2	109.84 (6)	C1^i^—C2—H2A	109.7
O3—Cr—O1	109.85 (8)	C1—C2—H2B	109.7
O4—Cr—O1	110.21 (8)	C1^i^—C2—H2B	109.7
O2—Cr—O1	108.02 (7)	H2A—C2—H2B	108.2
Cr—O1—Cr^i^	132.57 (11)	N1—C1—C2	111.03 (13)
C1—N1—H1A	109.5	N1—C1—H1D	109.4
C1—N1—H1B	109.5	C2—C1—H1D	109.4
H1A—N1—H1B	109.5	N1—C1—H1E	109.4
C1—N1—H1C	109.5	C2—C1—H1E	109.4
H1A—N1—H1C	109.5	H1D—C1—H1E	108.0
H1B—N1—H1C	109.5		

Symmetry code: (i) *x*, −*y*+1/2, *z*.

### Hydrogen-bond geometry (Å, º)

**Table:**

*D*—H···*A*	*D*—H	H···*A*	*D*···*A*	*D*—H···*A*
N1—H1*A*···O2^ii^	0.89	2.51	2.9326 (19)	110
N1—H1*A*···O3^iii^	0.89	2.12	2.9609 (19)	156
N1—H1*B*···O2^iv^	0.89	1.99	2.8168 (19)	154
N1—H1*C*···O4	0.89	2.17	2.955 (2)	147
N1—H1*C*···O2^v^	0.89	2.44	2.9844 (19)	120
C1—H1*D*···O3	0.97	2.51	3.405 (2)	153
C1—H1*E*···O2^ii^	0.97	2.59	3.176 (2)	119

Symmetry codes: (ii) *x*, *y*, *z*−1; (iii) *x*+1/2, *y*, −*z*+1/2; (iv) −*x*+1, −*y*, −*z*+1; (v) −*x*+3/2, −*y*, *z*−1/2.
